# Reactive Oxygen Species Regulate Protrusion Efficiency by Controlling Actin Dynamics

**DOI:** 10.1371/journal.pone.0041342

**Published:** 2012-08-02

**Authors:** Nicolas Taulet, Violaine D. Delorme-Walker, Céline DerMardirossian

**Affiliations:** Department of Immunology and Microbial Science, The Scripps Research Institute, La Jolla, California, United States of America; University of Edinburgh, United Kingdom

## Abstract

Productive protrusions allowing motile cells to sense and migrate toward a chemotactic gradient of reactive oxygen species (ROS) require a tight control of the actin cytoskeleton. However, the mechanisms of how ROS affect cell protrusion and actin dynamics are not well elucidated yet. We show here that ROS induce the formation of a persistent protrusion. In migrating epithelial cells, protrusion of the leading edge requires the precise regulation of the lamellipodium and lamella F-actin networks. Using fluorescent speckle microscopy, we showed that, upon ROS stimulation, the F-actin retrograde flow is enhanced in the lamellipodium. This event coincides with an increase of cofilin activity, free barbed ends formation, Arp2/3 recruitment, and ERK activity at the cell edge. In addition, we observed an acceleration of the F-actin flow in the lamella of ROS-stimulated cells, which correlates with an enhancement of the cell contractility. Thus, this study demonstrates that ROS modulate both the lamellipodium and the lamella networks to control protrusion efficiency.

## Introduction

Reactive oxygen species (ROS), including superoxide anion (O^2−^), hydrogen peroxide (H_2_O_2_), and singlet oxygen, are generated as byproducts of biological reactions catalyzed by oxidative enzymes such as xanthine oxidase, cytochrome P-450, mitochondrial oxidases and NADPH oxidases [1], [2], [3], [4]. ROS have been identified as major contributors to biological damage in organisms, acting to irreversibly alter or destroy the function of target molecules or membranes [5]. However, an appropriate or normal production of ROS has been demonstrated to function as an important signaling component and to play a major role in the host defense against bacterial and fungal pathogens [6].

Interestingly, ROS have emerged as important regulators of cell motility. Indeed, ROS derived from NADPH oxidase control colon adenocarcinoma cell migration [7], [8] and studies in vascular smooth muscle cells (VSMC) and in endothelial cells have shown that growth factors-stimulated migration required ROS generation [9], [10]. Conversely, the inhibition of ROS production by a NADPH oxidase inhibitor diphenylene iodonium (DPI) or by deletion of Nox1, a member of the NADPH oxidase family, has been reported to reduce the speed and directionality of migrating cells [8], [11]. Recent works in zebrafish revealed the existence of a tissue-scale gradient of H_2_O_2_ induced by wounding and required for leukocyte migration toward the wound, highlighting the role of H_2_O_2_ as an important signal to direct cell motility [12].

Migrating cells respond to chemoattractant gradients by adopting a polarized morphology, with their leading edge oriented in the direction of the gradient. Leading edge protrusion is thought to initiate migration and set the direction of movement. Cells with weak polarity lose the ability to protrude in a single direction, resulting in random trajectories and reduced migration speed. Protrusion of the membrane is tightly coupled to actin polymerization, both through the elongation of pre-existing filaments and by nucleation of new filaments. Substantial evidence has shown that the Arp2/3 complex mediates new actin nucleation and branching observed in lamellipodia [13], although other data have implicated the action of formins [14] and filamin [15]. Other major regulators of leading edge actin dynamics are cofilin and ADF [16]. Cofilin/ADF act both to increase the number of free barbed ends available for actin elongation via their severing function, and to enhance the turnover of existing filaments necessary for actin remodeling via their depolymerizing function.

Previous works in epithelial cells defined two dynamically, molecularly, and functionally distinct F-actin networks at the cell leading edge: the lamellipodium and the lamella [17]. The lamellipodium is defined by a fast treadmilling F-actin network [18], [19] characterized by a thin band of high F-actin polymerization at the leading edge, adjacent to a similarly thin band of strong disassembly. In contrast, the lamella is dominated by random patterns of alternating F-actin assembly and disassembly puntae and is characterized by a slow actin retrograde flow. Important molecular differences between the two F-actin networks have been revealed by pharmacological and biochemical analysis. The lamellipodium is enriched in Arp2/3 and cofilin, both responsible for the fast actin treadmilling [19]. The lamella, however, is composed of myosin II and tropomyosin that regulate the contractile machinery responsible for the F-actin retrograde flow in this region. Tropomyosin suppresses the interaction of cofilin with F-actin and blocks Arp2/3 nucleation [20]. Recent studies have shown that microinjection of skeletal muscle tropomyosin selectively depleted the lamellipodium from the cell protrusion [21]. Surprisingly, cells without a lamellipodium were still highly motile, thus suggesting that the lamellipodium plays more a role in directional sensing and persistence.

Interestingly, ROS can influence F-actin dynamics both directly and indirectly. On the one hand, direct oxidation of actin by ROS has been shown to affect polymerization. Treatment of actin in vitro with high concentrations of H_2_O_2_ decreased the polymerization and elongation rates of actin filaments [22]. Other studies have shown that migrating cells produced ROS at the membrane ruffles which in turn increased actin polymerization [11], [23]. On the other hand, cytoskeletal rearrangements could result from indirect regulation of actin dynamics by ROS, translated through redox-sensitive enzymes. As such, the oxidation of the low-molecular weight protein tyrosine phosphatase gives rise to a decrease in RhoA activity required for Rac-induced formation of membrane ruffles [24]. In addition, the ROS-dependent activation of the cofilin phosphatase slingshot-cofilin pathway via H_2_O_2_-mediated oxidation of 14-3-3zeta was shown to stimulate the formation of cofilin-actin rods [25].

Despite these findings, the precise effect of ROS on actin dynamics in vivo is still not clearly defined. The study presented here aimed at deciphering the molecular mechanisms controlling F-actin dynamics in migrating cells in response to H_2_O_2_ stimulation, which is critical to understand how ROS promote cell motility.

## Results

### H_2_O_2_ Regulates Cell Migration and Protrusion Dynamics in PtK1 Cells

**Figure 1:**
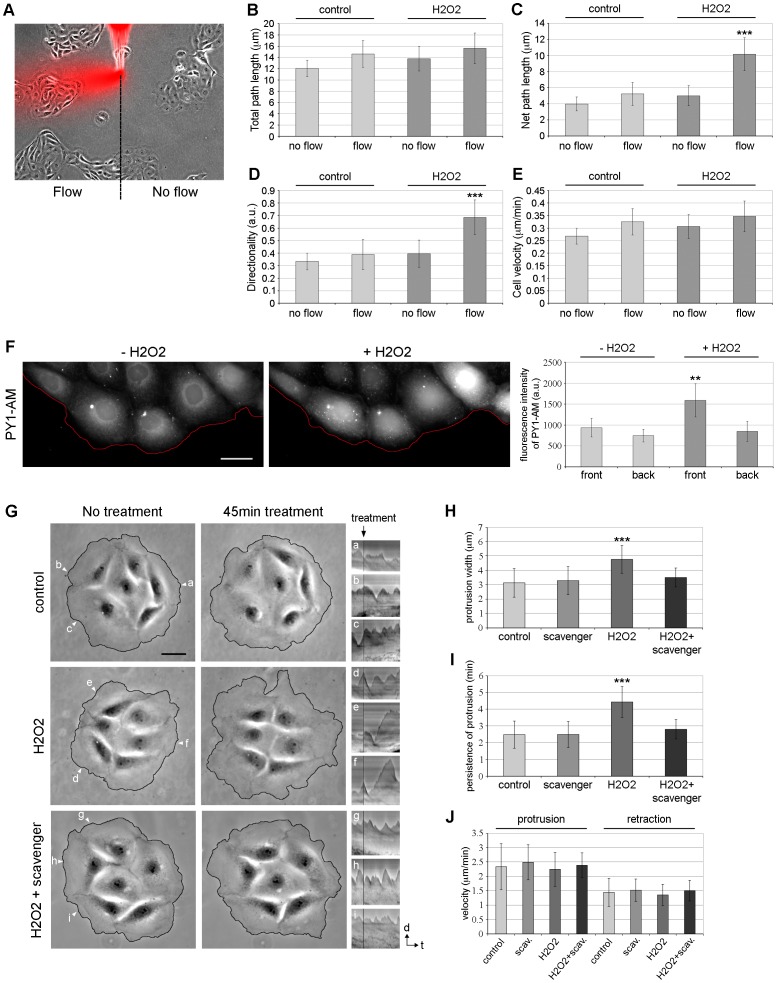
H_2_O_2_ regulates cell migration and protrusion dynamics in PtK1 cells. (A) Representative schema of the chemotaxis experiment. Using a microinjection system, PtK1 cells were exposed (flow) or not (no flow) to a constant flow of H_2_O (control) or 1.5 mM H_2_O_2_ mixed with rhodamine dextran (shown in red). The overlay of phase-contrast image and rhodamine dextran shows the direction of the flow. (B–E) Quantification of motility parameters of PtK1 cells exposed or not to a flow of H_2_O (control) or H_2_O_2_, including total path length (B), the total distance traversed by cells over time; net path length (C), the net distance that the cells traversed from the first to the last frame; directionality (D), the ratio of net to total path length; and cell velocity (E). The data result from n ≥38 cells analyzed for each condition. Error bars represent s.e.m. ***, p<0.001 compared to control (flow) and H_2_O_2_ (no flow). (F) PtK1 cells were incubated with the H_2_O_2_-sensitive probe PY1-AM (5 µM for 30 min) and then treated with H_2_O_2_ (500 µM for 20 min). One representative image is shown before and after H_2_O_2_ stimulation. Red lines highlight the leading edge of the cells. The scale bar is 30 µm. In the right panel, fluorescence intensity of PY1-AM was measured in cells located at the front and the back of PtK1 islands before and after H_2_O_2_ addition. Fluorescence intensity was averaged from 15 cells. Error bars represent s.e.m. **, p<0.01 compared to - H_2_O_2_ (front) and + H_2_O_2_ (back). (G) After starvation, PtK1 cells were incubated for 15 min in media only and 45 min with control media (+H_2_O) or media containing 500 µM H_2_O_2_ alone or in combination with 5 mM sodium pyruvate, a ROS scavenger. In this case, cells were pretreated with ROS scavenger for 30 min before the experiment. Phase-contrast images were taken from movies of each condition. Images of control (top row), H_2_O_2_- (center row) and H_2_O_2_+ROS scavenger-treated cells (bottom row) are shown before and after 45 min of treatment. The scale bar is 30 µm. White arrows highlight locations used to generate kymographs. Three representative kymographs of control (a, b and c), H_2_O_2_- (d, e and f) and H_2_O_2_+ROS scavenger-treated cells (g, h and i) are shown on the rightmost panels. Black lines on kymographs indicate when the cells were treated. The scale bar is indicated by black arrows corresponding to d = 5 µm and t = 15 min. (H–J) Protrusion width (H), persistence of protrusion (I) and protrusion/retraction velocities (J) resulting from the analysis of 25 control, ROS scavenger-, H_2_O_2_- and H_2_O_2_+ROS scavenger-treated cells and 125 kymographs per condition. Error bars represent s.e.m. ***, p<0.001 compared to control.

For our studies, we used mammalian PtK1 cells, derived from marsupial kidney epithelium, that have been shown to be ideal for imaging studies as they polarize in a large and thin cell front [17], [18], [21], [26], [27], [28]. We developed a chemotaxis assay to evaluate the effects of H_2_O_2_ on PtK1 cell migration. To this end, cells were exposed to a continuous flow of H_2_O or H_2_O_2_-containing solution from the tip of a microinjection needle for 45 min (Figure 1A-flow indicated in red). Cells present in the same imaging field but not in contact with the flow were used as control (Figure 1A-no flow). Analysis of individual cell tracks revealed that cells exposed to H_2_O_2_ displayed similar total path lengths, but increased net path lengths and directionality compared with control cells (Figure 1B–D). Moreover, cells exposed to H_2_O_2_ displayed similar migration rates compared to control cells (Figure 1E). These results indicate that PtK1 cells are more directional in presence of H_2_O_2_ and are consistent with data obtained in zebrafish in which H_2_O_2_ acts as an immediate signal that attracts leukocytes to a wound site [12]. During the chemotaxis assays, we observed that cells exposed to H_2_O_2_ seemed to protrude more than control cells. Thus, we investigated how H_2_O_2_ treatment affected protrusion dynamics of PtK1 cells. First, we wanted to confirm that H_2_O_2_ was incorporated within the cells following the treatment. To this end, PtK1 cells were incubated with the H_2_O_2_-specific probe PY1-AM (Peroxy-Yellow 1 Acetoxymethyl-ester) [29], [30], and treated with 500 µM of H_2_O_2_ for 20 min. Cells were visualized by epifluorescence microscopy before and after H_2_O_2_ addition (Figure 1F). We observed an increase in PY1-AM fluorescence intensity after stimulation with H_2_O_2_ which confirmed that H_2_O_2_ was present in these cells. Although the probe uptake was not homogenous in the entire cell island (Figure 1F), PY1-AM fluorescence intensity ratio after vs. before H_2_O_2_ addition was 1.69±0.36 at the front of the cell island and 1.13±0.15 at the back, thus indicating that H_2_O_2_ was incorporated in the cells at the edge of PtK1 islands before the cells located inside the islands (Figure 1F, right panel).

We next analyzed the protrusion behavior of cells in response to H_2_O_2_ using phase-contrast live-cell microscopy. PtK1 cells were starved overnight and then incubated either with control media (Figure 1G, top row) or with media containing 500 µM H_2_O_2_ (Figure 1G, center row) for 45 min (Videos S1, S2). Analysis of protrusion dynamics by kymographs (Figure 1G, right panels) revealed that cells treated with H_2_O_2_ exhibited a wider and more persistent protrusion (Figure 1H–I) compared to control cells. Analysis of protrusion velocity demonstrated that control cells or cells treated with H_2_O_2_ protruded faster than they retracted and had similar protrusion and retraction velocities (Figure 1J). Finally, we analyzed whether the effects of H_2_O_2_ on cell protrusion can be abrogated by sodium pyruvate, a well-characterized H_2_O_2_ scavenger [31]. Our data indicated that the protrusion parameters tested in cell treated with sodium pyruvate alone were not significantly different from control cells (Figure 1H–J). However, the addition of sodium pyruvate in cells treated with H_2_O_2_ induced a strong decrease in the protrusion width and persistence compared to H_2_O_2_-treated cells (Figure 1G, bottom row, Figure 1H–I, Video S3), no effect was detected on protrusion and retraction velocities compared to control or H_2_O_2_-treated cells (Figure 1J). Combined, these results indicate that H_2_O_2_ treatment induces the formation of a persistent protrusion in PtK1 cells.

### H_2_O_2_ Affects Actin Dynamics in PtK1 Cells

**Figure 2:**
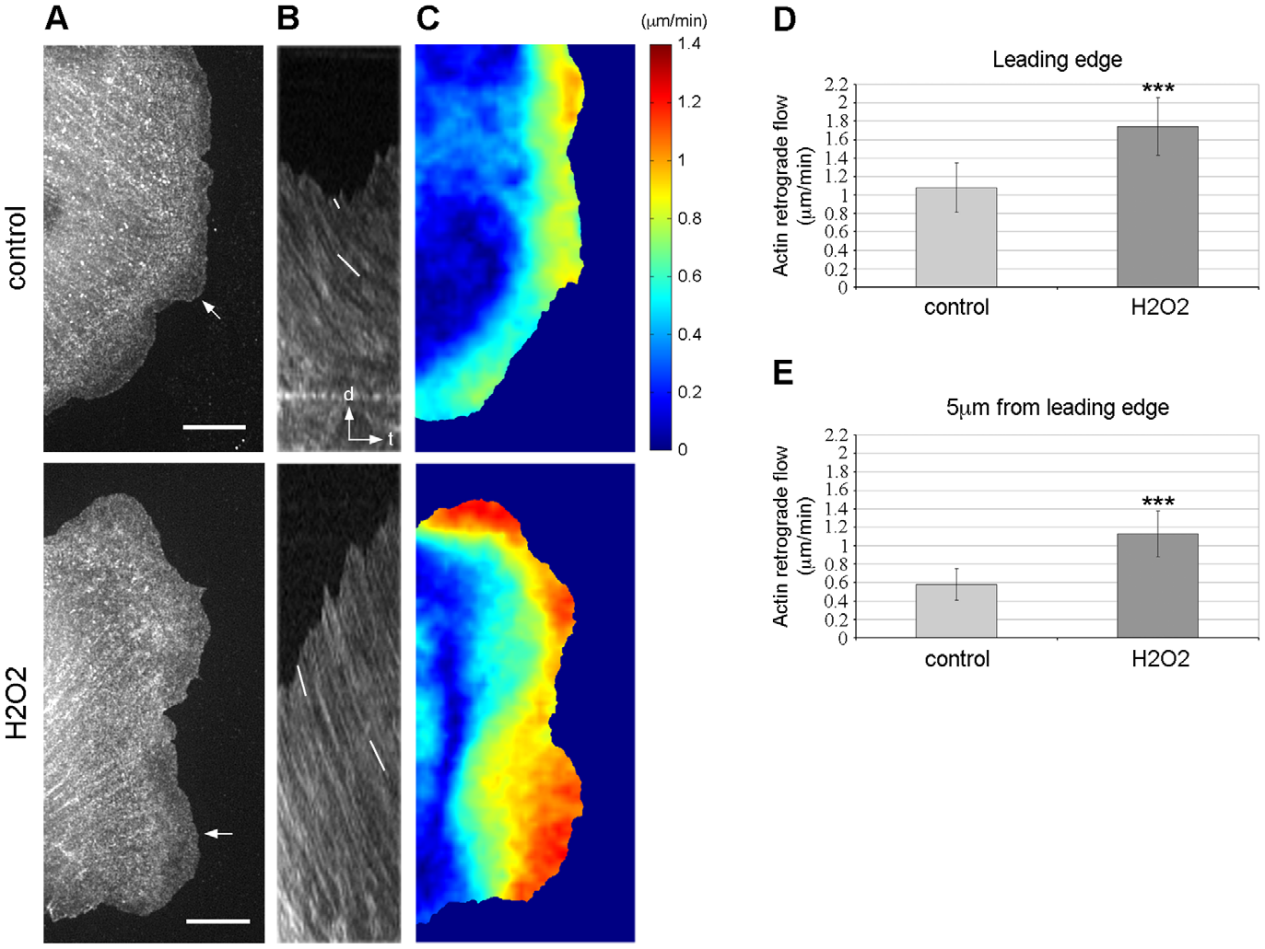
H_2_O_2_ modulates actin dynamics in PtK1 cells. (A) Single frames of actin fluorescent speckle time-lapse series of starved PtK1 control (top row) and treated with 500 µM H_2_O_2_ (bottom row). The scale bar is 10 µm. White arrows highlight the locations used to generate kymographs. (B) Kymographs of control and H_2_O_2_-treated cells depicted in (A). White lines indicate speckle translocation used to calculate flow velocities. (C) F-actin flow maps computed from quantitative FSM analysis of time-lapse movies of control and H_2_O_2_-treated cells. Flow rates are color coded, ranging from slow flow in dark blue to fast flow in red. Flow maps have been averaged over 60 frames, i.e., 10 min. (D and E) Average F-actin flow rates measured at the leading edge (D) and 5 µm from the leading edge (E) of control and H_2_O_2_-treated cells. n = 14 cells analyzed for control and H_2_O_2_ treatment. Error bars represent s.e.m. ***, p<0.001 compared to control.

Protrusive activity is driven by a tight regulation of the actin cytoskeleton at the front of the cells. To determine the effects of H_2_O_2_ treatment on actin dynamics at the leading edge of PtK1 cells, we used quantitative fluorescent speckle microscopy (qFSM) [32] (Figure 2). Cells were injected with X-rhodamine-labelled actin, then starved for 3 h, and FSM time-lapse series were acquired at 10 sec intervals for 10 min (Figure 2A and Videos S4, S5). Kymograph analysis of F-actin velocity indicated that, in control cells, F-actin underwent a slow retrograde flow in the lamella (0.6 µm/min, Figure 2B, top panel, and Figure 2E) and a faster retrograde flow in the lamellipodium (1.1 µm/min, Figure 2B, top panel, and Figure 2D), as previously observed in other studies [17], [18], [21], [26]. In comparison to control cells, cells treated with H_2_O_2_ displayed a fast F-actin retrograde flow not only at the leading edge but also throughout the entire protrusion (Figure 2B, bottom panel). Quantification of the F-actin flow rates using kymographs indicated that cells treated with H_2_O_2_ had an increase in the flow speed at the leading edge by ∼1.6 fold (1.75 µm/min) compared to control cells (1.1 µm/min) (Figure 2D), while at 5 µm from the leading edge, the retrograde flow was increased by ∼1.8 fold (1.1 µm/min) compared to unstimulated control cells (0.6 µm/min) (Figure 2E). These data were supported by spatially resolved maps of F-actin flow speed which showed an increase in the flow speed (Figure 2C) and a widening of the region of fast F-actin flow at the leading edge of H_2_O_2_-treated cells compared with untreated cells (Figure 2C). Together, these results indicate that H_2_O_2_ regulates actin dynamics in PtK1 cells and induces the formation of a wide region of fast actin retrograde flow.

### H_2_O_2_ Induces Cofilin Activation and Increases the Formation of Polymerization-competent Free Barbed Ends at the Cell Edge in PtK1

**Figure 3:**
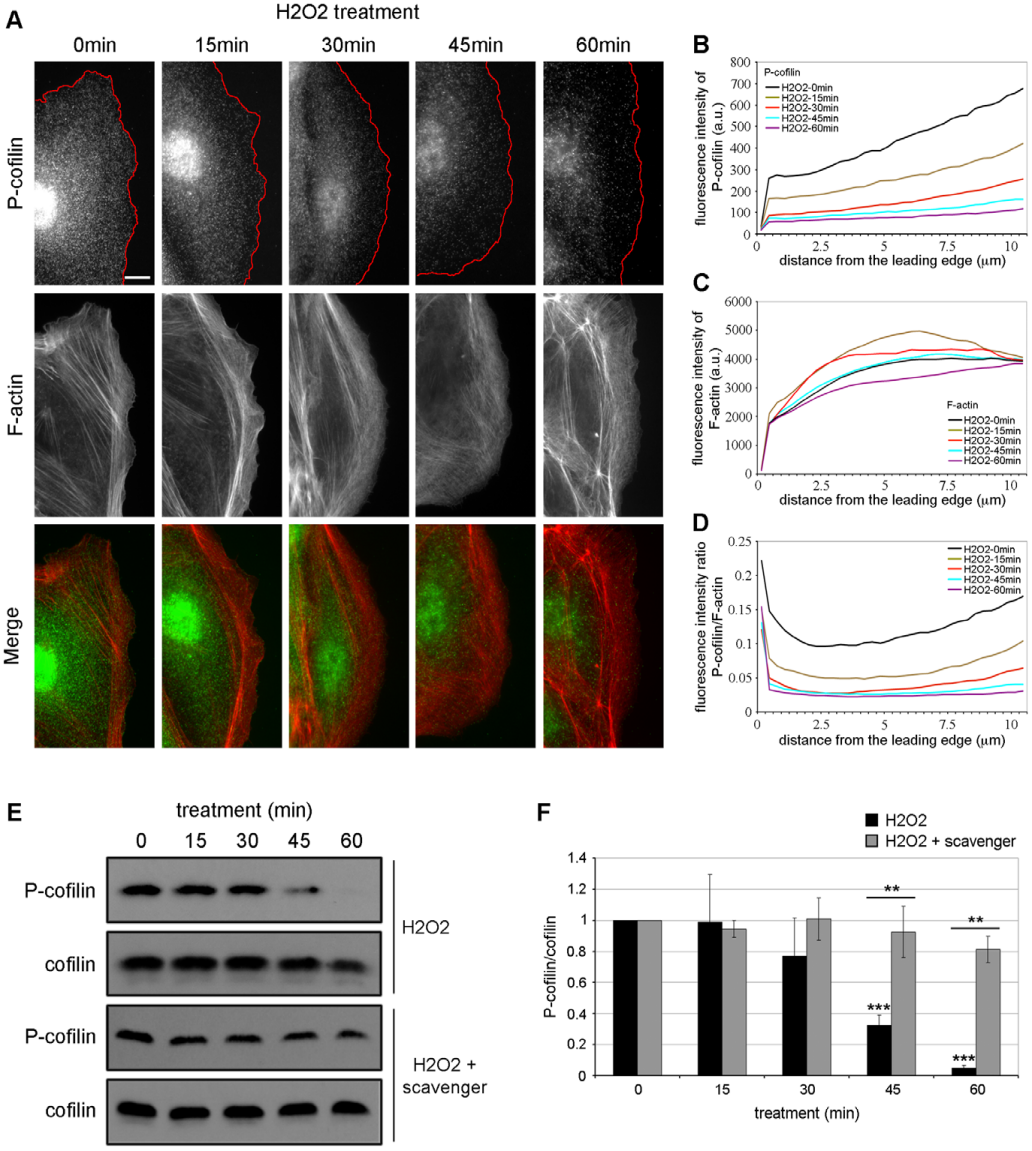
Cofilin is activated upon H_2_O_2_ stimulation in PtK1 cells. (A) Immunolocalization of phosphorylated cofilin (P-cofilin, green) and F-actin phalloidin staining (red) in starved PtK1 cells treated with 500 µM H_2_O_2_ for the indicated times. The scale bar is 10 µm. Red lines highlight the leading edge of the cells. (B and C) Fluorescence intensity of P-cofilin (B) and F-actin (C) in cells treated with 500 µM H_2_O_2_, measured from the cell edge (0 µm) into the cell center (10 µm). (D) P-cofilin/F-actin fluorescence intensity ratio in cells treated with 500 µM H_2_O_2_, measured from the cell edge (0 µm) into the cell center (10 µm). In (B)–(D), the data shown represent one experiment and are averaged from at least 18 cells for each condition. The experiment was repeated three times with similar results (Figure S2A). (E) Cell lysates from starved PtK1 cells treated with 500 µM H_2_O_2_ alone or in combination with ROS scavenger (5 mM) for 0-15-30-45-60 min were immunoblotted with antibodies against P-cofilin and cofilin. In (F), the graph represents the averaged P-cofilin/cofilin values. Data are from six and three independent experiments for H_2_O_2_ and H_2_O_2_+ROS scavenger, respectively. Error bars represent s.e.m. ***, p<0.001 compared to 0 min H_2_O_2_ and **, p<0.01 compared to 45–60 min H_2_O_2_.

To investigate whether the F-actin dynamics phenotype observed in H_2_O_2_-treated cells resulted from a regulation of the lamellipodium and/or the lamella, we next examined the distribution of the molecular markers of both regions. We first examined the localization of endogenous inactive phosphorylated cofilin (P-cofilin), a lamellipodium marker, in cells unstimulated or treated with 500 µM of H_2_O_2_ for various periods of time. In control cells (unstimulated), P-cofilin localized in diffuse punctae throughout the cell edge and within the cell body (Figure 3A). Upon H_2_O_2_ stimulation, we observed a gradual decrease of P-cofilin from the leading edge throughout the protrusion (Figure 3A). Quantification of P-cofilin relative to F-actin fluorescence intensities from the leading edge toward the cell center indicated a ∼1.9 fold decrease in P-cofilin by 15 min that reached a 3 to 4 fold decrease by 30–60 min at the cell edge, as compared to unstimulated cells (Figure 3B–D and Figures S1–S2A). Quantification of F-actin fluorescence intensity revealed a slight increase of F-actin at the leading edge of cells treated with H_2_O_2_ for 15–30 min (Figure 3C). We next analyzed by western blot the P-cofilin and cofilin expression levels in total cell lysates from cells treated or not with H_2_O_2_. As shown in Figure 3E, the level of P-cofilin was slightly decreased after 30 min of H_2_O_2_ treatment, followed by a significant decreased at 45–60 min upon stimulation, as previously observed [25]. The level of cofilin was similar in all conditions (Figure 3E). Quantification of the ratio P-cofilin/cofilin revealed a decrease of 70% and 95% in cofilin phosphorylation after 45 and 60 min of H_2_O_2_ stimulation, respectively, compared to untreated cells (Figure 3F). We next examined the effect of sodium pyruvate on the level of P-cofilin upon H_2_O_2_ stimulation. As shown in Figure 3E–F, the presence of ROS scavenger strongly abrogated the cofilin dephosphorylation observed upon H_2_O_2_ stimulation in PtK1 cells, thus confirming the specific effect of H_2_O_2_ on cofilin activation. We previously observed that H_2_O_2_ was incorporated in the cells located at the edge of PtK1 islands before the cells present inside the islands (Figure 1F). This suggests that cofilin was activated first in the cells located at the edge of cell islands, which were analyzed by immunofluorescence. This could explain the cofilin activation delay observed between immunofluorescence and western blot, in which the whole population of cells was analyzed. Taken together, these data indicate that cofilin is activated upon H_2_O_2_ stimulation in PtK1 cells and that this activation occurs at the cell edge starting at 15 min after treatment.

**Figure 4:**
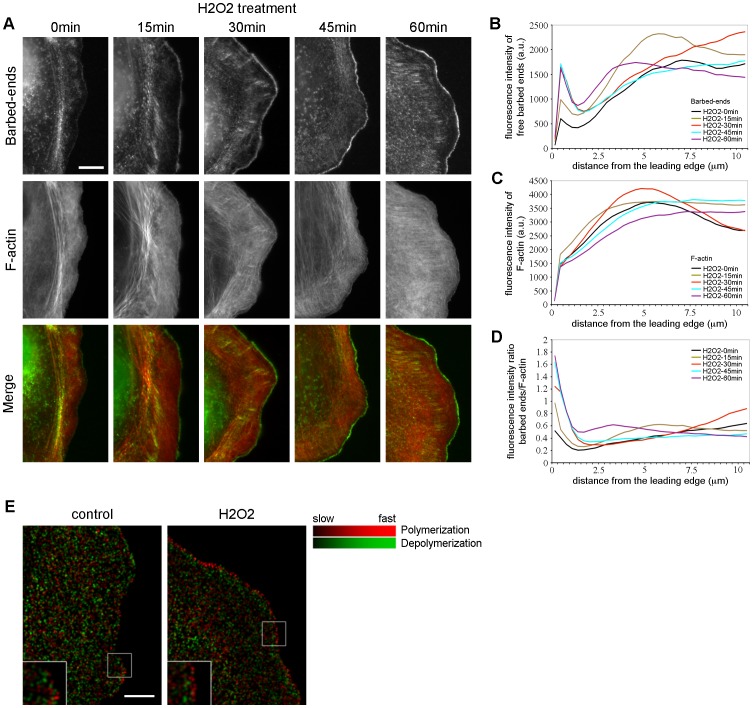
H_2_O_2_ increases the formation of free barbed ends and F-actin turnover. (A) Free barbed end actin incorporation (green) and F-actin phalloidin staining (red) in starved PtK1 cells treated with 500 µM H_2_O_2_ for the indicated times. The scale bar is 10 µm. (B and C) Fluorescence intensity of free barbed end actin incorporation (B) and F-actin (C) in cells treated with 500 µM H_2_O_2_, measured from the cell edge (0 µm) into the cell center (10 µm). (D) Fluorescence intensity ratio of free barbed end actin incorporation relative to F-actin in cells treated with 500 µM H_2_O_2_, measured from the cell edge (0 µm) into the cell center (10 µm). In (B)-(D), the data shown represent one experiment and are averaged from at least 16 cells for each condition. The experiment was repeated four times with similar results (Figure S2B). (E) F-actin turnover maps computed from quantitative FSM time-lapse movies of starved PtK1 cells treated or not with 500 µM H_2_O_2_. Turnover maps depict F-actin polymerization (red) and depolymerization (green) rates. Maps have been averaged over 6 frames, i.e., 1 min. The scale bar is 10 µm. Boxed regions are magnified in the bottom left of each panel. n = 14 cells analyzed for control and H_2_O_2_ treatment.

Since cofilin is thought to supply polymerization-competent free barbed filament ends to the lamellipodium [33], we further analyzed the localization and density of free barbed ends upon H_2_O_2_ treatment in PtK1 cells. In starved control cells (unstimulated), few free barbed ends were observed at the cell edge (Figure 4A). In contrast, the concentration of free barbed ends was slightly increased after 15 min of H_2_O_2_ treatment and dramatically increased after 30–60 min in a narrow rim along the leading edge (Figure 4A). Quantification of the fluorescence intensity of barbed ends to F-actin ratio confirmed that free barbed ends were increased at the cell edge by ∼1.8 fold after 15 min and by ∼3 fold after 45 and 60 min of treatment (Figure 4B–D and Figure S2B). These data were consistent with the increase of cofilin activity we observed previously (Figure 3E–F). As described above, quantification of the fluorescence intensity of F-actin slightly increased after H_2_O_2_ stimulation (Figure 4C). Free barbed filament ends localized in a 1.3 µm region along the leading edge (Figure 4B–D). The width of the barbed ends area was not significantly modified upon H_2_O_2_ treatment. To further support these results, we analyzed the spatial organization of F-actin assembly-disassembly rates in PtK1 cells treated or not with H_2_O_2_. The lamellipodium is typically defined by a narrow band of polymerization adjacent to a narrow band of depolymerization [17]. Actin turnover maps, obtained from FSM time-lapse series, revealed that control cells had a mixed region of both polymerization (bright red punctae) and depolymerization (bright green punctae) events along the cell edge (Figure 4E). In starved cells, the lamellipodium is so thin (Figure 2B, top panel) that the integration of all the frames abolished the distinction between local assembly/disassembly bands. In contrast, H_2_O_2_-treated cells exhibited a wider band of strong polymerization along the leading edge juxtaposed to a similar band of rapid F-actin depolymerization (Figure 4E, bright red and bright green, respectively), indicative of a fast treadmilling lamellipodium. Altogether, our data suggest that H_2_O_2_ treatment induces the formation of polymerization-competent free barbed ends and increases F-actin turnover at the edge of PtK1 cells.

### H_2_O_2_ Induces Arp2/3 Recruitment and ERK Activation at the Leading Edge of PtK1 Cells

**Figure 5:**
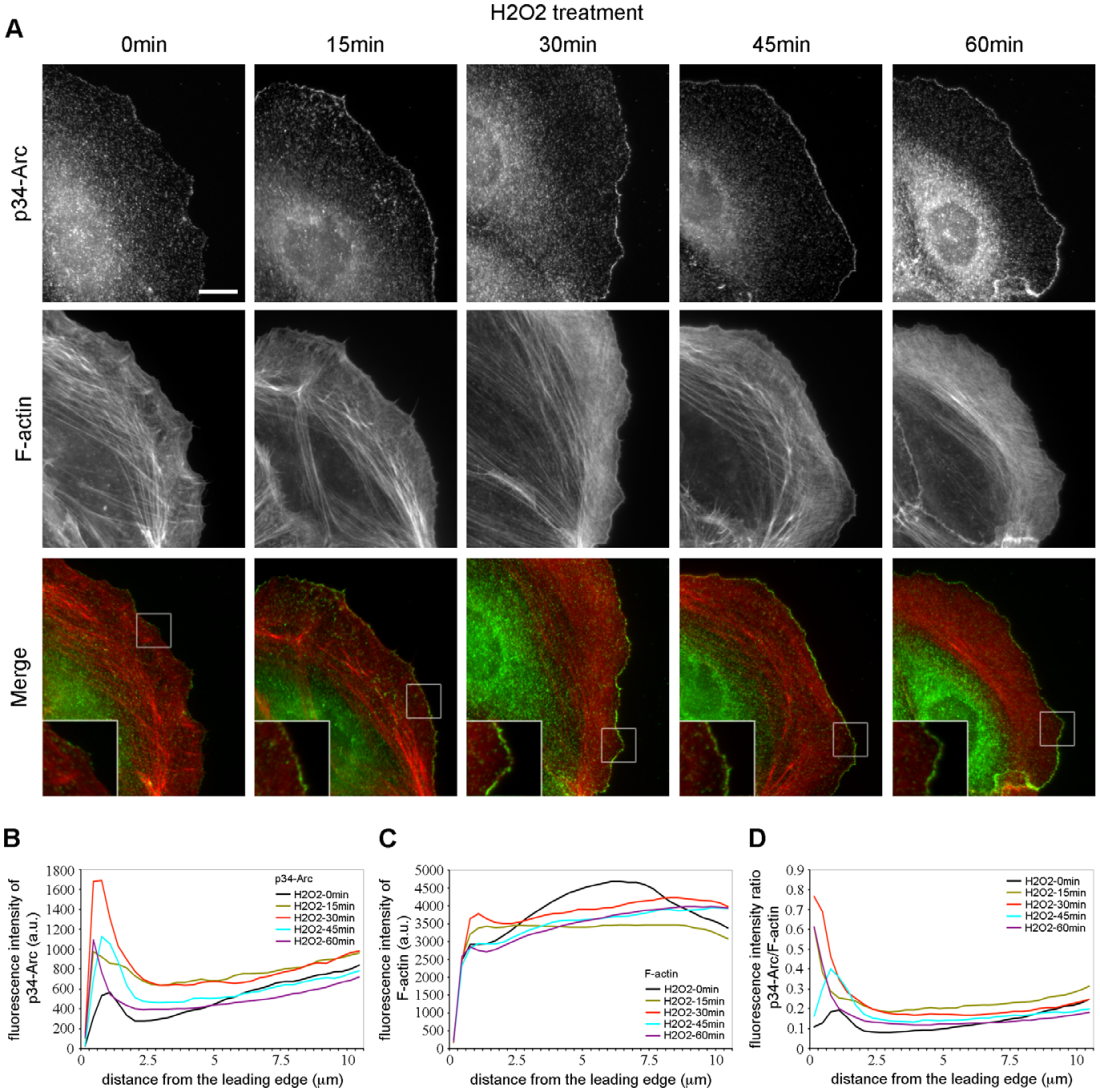
H_2_O_2_ induces Arp2/3 recruitment at the leading edge of PtK1 cells. (A) Immunolocalization of p34-Arc (green) and F-actin phalloidin staining (red) in starved PtK1 cells treated with H_2_O_2_ 500 µM for the indicated times. The scale bar is 10 µm. Boxed regions are magnified in the bottom left of the Merge panel. (B and C) Fluorescence intensity of p34-Arc (B) and F-actin (C) in cells treated with 500 µM H_2_O_2_, measured from the cell edge (0 µm) into the cell center (10 µm). (D) p34-Arc/F-actin fluorescence intensity ratio in cells treated with 500 µM H_2_O_2_, measured from the cell edge (0 µm) into the cell center (10 µm). In (B)–(D), the data shown represent one experiment and are averaged from at least 12 cells for each condition. The experiment was repeated three times with similar results (Figure S2C).

Several studies suggest a synergy between the severing activity of cofilin and the branching activity of Arp2/3 to regulate actin dynamics in the lamellipodium [34], [35], [36]. The Arp2/3 complex drives leading edge protrusion by nucleating actin monomers to assemble a dense meshwork of short branched filaments [37]. Thus, we analyzed the distribution of the Arp2/3 complex in PtK1 cells stimulated with H_2_O_2_ for up to 60 min. In unstimulated control cells, p34-Arc, a subunit of the Arp2/3 complex, was detected throughout the cell (Figure 5A). We observed a dramatic increase of p34-Arc recruitment to the cell edge after H_2_O_2_ treatment (Figure 5A). Quantification of the fluorescence intensity from the leading edge toward the cell center confirmed that p34-Arc increased in density at the leading edge of H_2_O_2_-treated cells as soon as 15 min after stimulation, and with the highest concentration at 30 min (∼4 fold above control) (Figure 5B–D and Figure S2C). Thus, H_2_O_2_ treatment induced the recruitment of the Arp2/3 complex at the leading edge of PtK1 cells.

**Figure 6:**
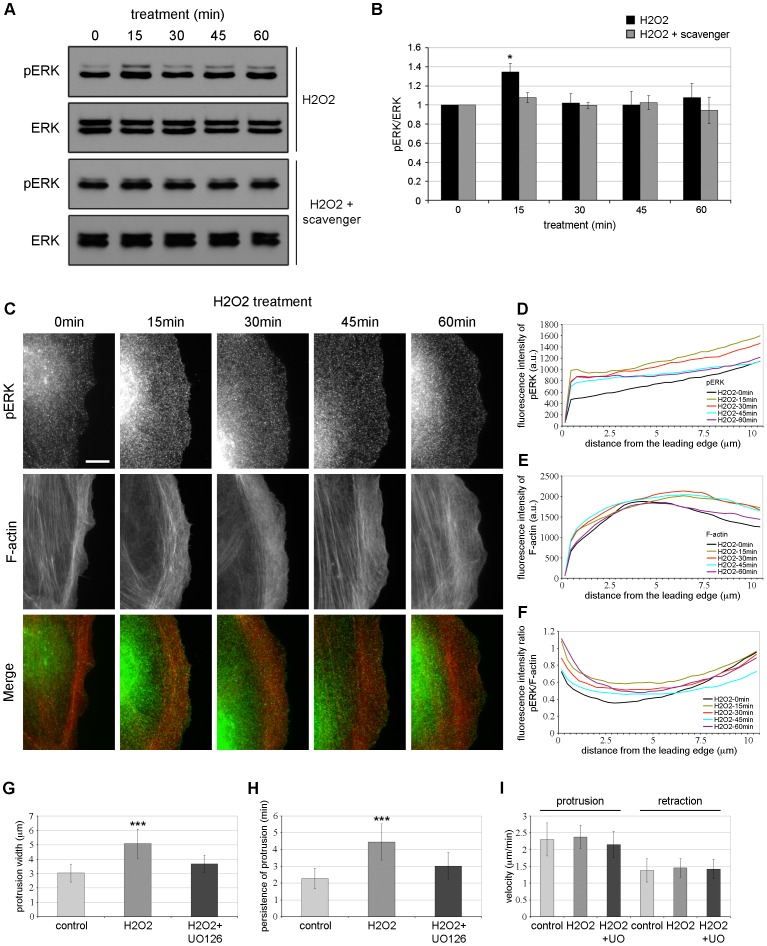
ERK is activated in response of H_2_O_2_ and contributes to H_2_O_2_-induced protrusion dynamics. (A) Cell lysates from starved PtK1 cells treated with 500 µM H_2_O_2_ alone or in combination with ROS scavenger (5 mM) for 0-15-30-45-60 min were immunoblotted with antibodies against pERK and ERK. In (B), the graph represents the averaged normalized pERK values. Data are from four and three independent experiments for H_2_O_2_ and H_2_O_2_+ROS scavenger, respectively. Error bars represent s.e.m. *, p<0.05 compared to 0 min H_2_O_2_ and 15 min H_2_O_2_+scavenger. (C) Immunolocalization of phosphorylated ERK (pERK, green) and F-actin phalloidin staining (red) in starved PtK1 cells treated with H_2_O_2_ 500 µM for the indicated times. The scale bar is 10 µm. (D and E) Fluorescence intensity of pERK (D) and F-actin (E) in cells treated with H_2_O_2_ 500 µM, measured from the cell edge (0 µm) into the cell center (10 µm). (F) pERK/F-actin fluorescence intensity ratio in cells treated with H_2_O_2_ 500 µM, measured from the cell edge (0 µm) into the cell center (10 µm). In (D)–(F), the data shown represent one experiment and are averaged from at least 13 cells for each condition. The experiment was repeated three times with similar results (Figure S2D). Protrusion width (G), persistence of protrusion (H) and protrusion/retraction velocities (I) in starved Ptk1 cells incubated for 45 min with control media (+DMSO) or media containing 500 µM H_2_O_2_ alone or in combination with UO126, a MEK inhibitor. In (G)–(I), the data shown result from the analysis of at least 23 cells and 115 kymographs per condition. Error bars represent s.e.m. ***, p<0.001 compared to control and H_2_O_2_+UO126.

Interestingly, a recent study has shown a link between the regulation of Arp2/3 and ERK (extracellular signal-regulated kinase) signaling. Indeed, the activity of ERK specifically regulates protrusion initiation through the phosphorylation of the WAVE2 regulatory complex, which is required for its functional binding and activation of Arp2/3 [38]. Thus, we analyzed by western blot the activity of ERK in PtK1 cells treated with 500 µM of H_2_O_2_ for 0 to 60 min. Of note, the western blot with pERK antibody (Figure 6A) showed two bands at 42 and 44 kDa. This antibody recognized both pERK1 (top band, 44 kDa) and pERK2 (bottom band, 42 kDa). Upon H_2_O_2_ stimulation, pERK level increased at 15 min compared to control cells and by 30 min the level of pERK decreased to the basal level of control cells (Figure 6A). The ERK protein level did not change during the time of stimulation (Figure 6A). Quantification of the ratio pERK/ERK indicated a notable ∼1.35 fold increase in ERK phosphorylation after 15 min of H_2_O_2_ stimulation while no significant activation was detected at 30–60 min compared to untreated cells (Figure 6B). Conversely, addition of ROS scavenger to the culture media abrogated the ERK activation observed in the presence of H_2_O_2_ alone (Figure 6A–B). We next examined the localization of active phosphorylated ERK in starved PtK1 cells stimulated with H_2_O_2_. In unstimulated condition, pERK was distributed throughout the cell (Figure 6C). After 15 min of treatment, pERK signal was increased not only at the cell edge but also throughout the entire protrusion and at 30–60 min, pERK signal decreased within the cell body but was still detected in punctae at the cell edge (Figure 6C). Quantification of the pERK/F-actin fluorescence intensity ratio confirmed a significant increase of 1.5 fold in pERK localization at the edge of H_2_O_2_-treated cells compared to control cells (Figure 6D–F and Figure S2D). Of note, we observed that other MAPK, such as p38 and JNK, were activated during H_2_O_2_ stimulation (Figure S3F–G). However, since ERK signaling has been involved in the regulation of protrusion dynamics, we decided to further investigate its role in response to H_2_O_2_. To this end, we treated the cells with a MEK inhibitor, UO126. We analyzed by western blot the pERK and ERK expression levels in total cell lysates from cells treated with UO126 and stimulated with H_2_O_2_. As shown in Figure S3A, the level of pERK was completely decreased after 15 min of H_2_O_2_ treatment and a slight increase was observed at 60 min. The level of ERK was similar in all conditions (Figure S3A). We next analyzed the protrusion dynamics as described above. We observed that inhibition of ERK in H_2_O_2_-treated cells significantly decreased protrusion width and persistence compared to cells treated with H_2_O_2_ alone (Figure 6G–I and Video S6). Finally, we analyzed whether the inhibition of ERK in cells stimulated with H_2_O_2_ affected the localization of p34-Arc (Figure S3B–E). The cells were stimulated for 30 min, a time point shown to induce a major peak of p34-Arc accumulation at the cell leading edge (Figure 5). Interestingly, ERK inhibition decreased p34-Arc recruitment to the cell edge of H_2_O_2_-treated cells compared to cells treated with H_2_O_2_ alone (Figure S3B–C). Quantification of p34-Arc relative to F-actin fluorescence intensities indicated a ∼1.4 fold decrease in p34-Arc as compared to cells treated with H_2_O_2_ alone (Figure S3E and Figure S2H). Analysis of F-actin fluorescence intensity revealed no effect on F-actin at the leading edge of cells treated with H_2_O_2_ for 30 min in presence or absence of U0126 (Figure S3D). Collectively, our results confirm the involvement of ERK in H_2_O_2_-induced protrusion dynamics and indicate that ERK signaling partially mediates the recruitment of Arp2/3 to the edge of cells stimulated with H_2_O_2_.

### H_2_O_2_ Regulates the Distribution of Lamella Markers and the Contractile Machinery in PtK1 Cells

**Figure 7:**
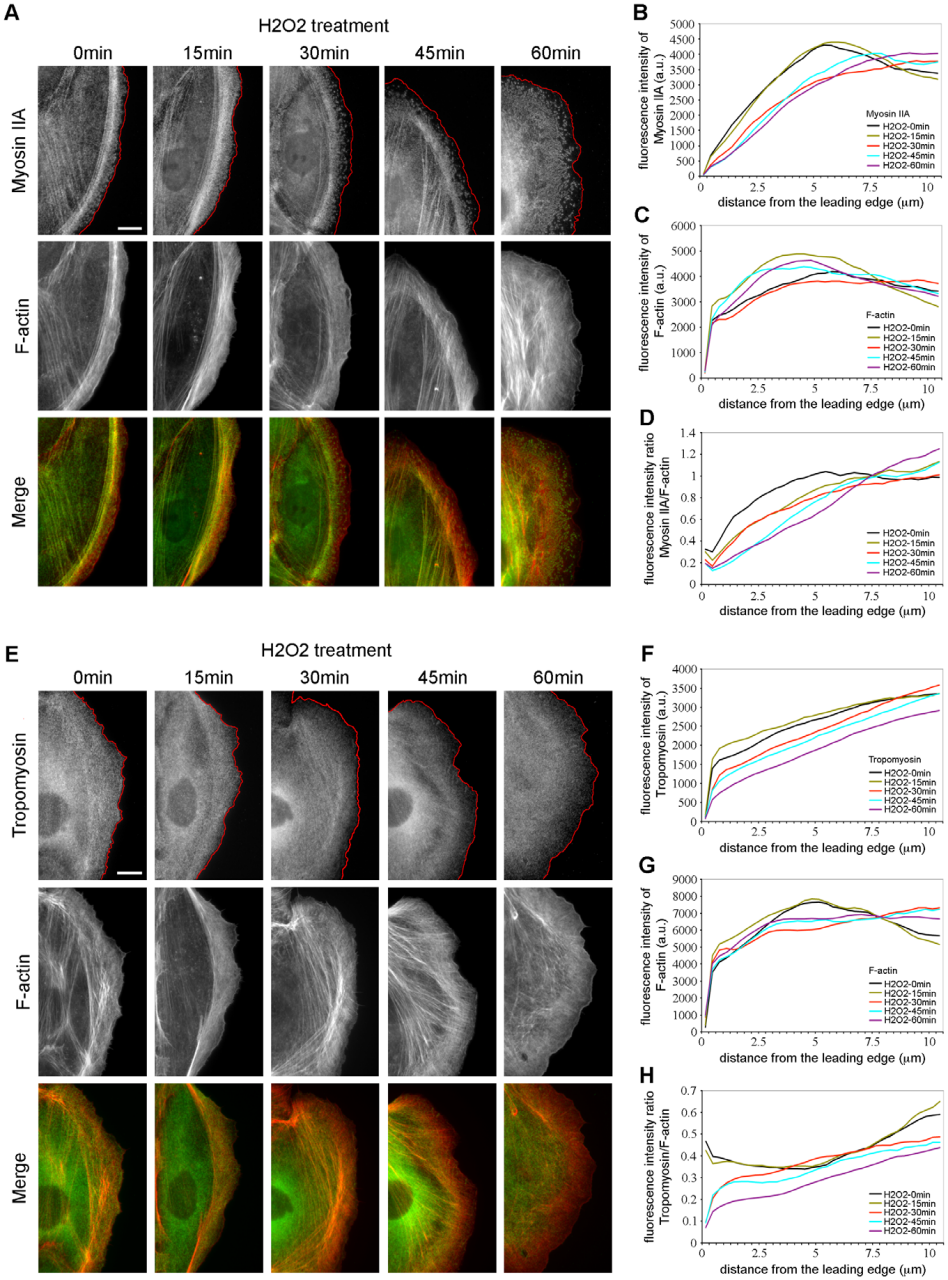
H_2_O_2_ depletes myosin IIA and tropomyosin from the leading edge. (A) Immunolocalization of Myosin IIA (green) and F-actin phalloidin staining (red) in starved PtK1 cells treated with H_2_O_2_ 500 µM for the indicated times. The scale bar is 10 µm. Red lines highlight the leading edge of the cells. (B and C) Fluorescence intensity of Myosin IIA (B) and F-actin (C) in cells treated with H_2_O_2_ 500 µM, measured from the cell edge (0 µm) into the cell center (10 µm). (D) Myosin IIA/F-actin fluorescence intensity ratio in cells treated with H_2_O_2_ 500 µM, measured from the cell edge (0 µm) into the cell center (10 µm). In (B)–(D), the data shown represent one experiment and are averaged from at least 14 cells for each condition. The experiment was repeated three times with similar results (Figure S2E). (E) Immunolocalization of Tropomyosin (green) and F-actin phalloidin staining (red) in starved PtK1 cells treated with H_2_O_2_ 500 µM for the indicated times. The scale bar is 10 µm. Red lines highlight the leading edge of the cells. (F and G) Fluorescence intensity of Tropomyosin (F) and F-actin (G) in cells treated with H_2_O_2_ 500 µM, measured from the cell edge (0 µm) into the cell center (10 µm). (H) Tropomyosin/F-actin fluorescence intensity ratio in cells treated with 500 µM H_2_O_2_, measured from the cell edge (0 µm) into the cell center (10 µm). In (F)–(H), the data shown represent one experiment and are averaged from at least 13 cells for each condition. The experiment was repeated three times with similar results (Figure S2F).

Using FSM, we showed that H_2_O_2_ treatment dramatically increased the F-actin retrograde flow from the leading edge to further inside the protrusion (Figure 2). This phenotype could result from (i) a widening of the lamellipodium, (ii) an increase in F-actin retrograde flow in the lamella, or (iii) an increase in both the lamellipodium and the lamella. To differentiate between these hypotheses, we first examined by immunofluorescence whether the distribution of lamella markers was affected upon H_2_O_2_ treatment. We analyzed the localization of myosin IIA and tropomyosin, which are exclusively associated with the lamella and involved in the regulation of the contractile machinery [17], [21]. In control cells (0 min H_2_O_2_), myosin IIA was excluded from the lamellipodium and appeared in a gradient of punctae within the lamella (Figure 7A). Upon H_2_O_2_ treatment, myosin IIA was depleted farther from the cell edge at 30–60 min after stimulation (Figure 7A). These observations were confirmed by quantification of the fluorescence intensity of myosin IIA (Figure 7B), measured from the cell edge into the cell center. The ratio myosin IIA/F-actin (Figure 7C–D and figure S2E) indicated that in control cells, myosin IIA was localized at ∼0.8 µm from the cell edge, while after 30 min of treatment, myosin IIA was depleted in a ∼1.8 µm area from the leading edge and decreased by 1.4 fold in intensity compared to control cells. At 45 and 60 min after addition of H_2_O_2_, myosin IIA was reduced in a 3 to 3.5 µm region from the cell edge, respectively. These data indicate that H_2_O_2_ displaced myosin IIA from the cell edge. We next analyzed by immunofluorescence the localization of high-molecular weight isoforms of tropomyosin, which regulate myosin II interaction with F-actin as characterized in skeletal muscle [39]. In control cells or cells treated with H_2_O_2_ for 15 min, we observed that tropomyosin localized throughout the cell (Figure 7E). After 30–60 min of stimulation, tropomyosin was drastically reduced from the cell edge (Figure 7E). Quantification of the fluorescence intensity ratio of tropomyosin/F-actin from the leading edge toward the cell center confirmed our observation and indicated that at 30–45 and 60 min of stimulation, tropomyosin was reduced by ∼1.8 to 2.7 fold at the leading edge of PtK1 cells compared to untreated cells and was depleted in the first micrometers adjacent to the cell edge (Figure 7F–H and Figure S2F). Thus, upon H_2_O_2_ treatment, myosin IIA and tropomyosin were depleted much farther from the cell edge compared to unstimulated cells, suggesting a widening of the lamellipodium.

**Figure 8:**
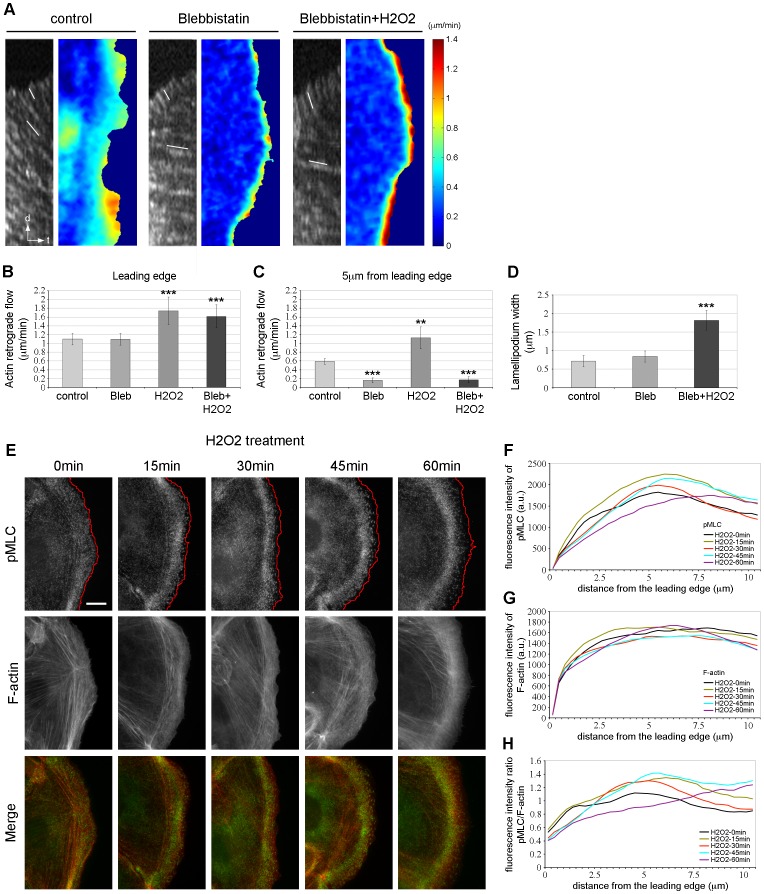
H_2_O_2_ regulates the contractile machinery of the lamella. (A) Kymographs and F-actin flow maps computed from quantitative FSM analysis of time-lapse movies of control, blebbistatin (50 µM) and blebbistatin (50 µM)+H_2_O_2_ 500 µM-treated cells. White lines on kymographs indicate speckle translocation used to calculate flow velocities. Flow rates are color coded, ranging from slow flow in dark blue to fast flow in red. Flow maps have been averaged over 30 frames, i.e., 5 min. (B and C) Average F-actin flow rates measured at the leading edge (B) and 5 µm from the leading edge (C) of control, blebbistatin, H_2_O_2_ and blebbistatin + H_2_O_2_-treated cells. n ≥7 cells from at least four independent experiments. Five kymographs/cell were analyzed for each condition. Error bars represent s.e.m. ***, p<0.001 compared to control and blebbistatin (B). **, p<0.001 compared to control and ***, p<0.001 compared to control and H_2_O_2_ (C). (D) Average lamellipodium width of control, blebbistatin and blebbistatin + H_2_O_2_-treated cells. n≥7 cells from at least four independent experiments. Five kymographs/cell were analyzed for each condition. Error bars represent s.e.m. ***, p<0.001 compared to control and blebbistatin. (E) Immunolocalization of phosphorylated MLC (pMLC, green) and F-actin phalloidin staining (red) in starved PtK1 cells treated with H_2_O_2_ 500 µM for the indicated times. The scale bar is 10 µm. Red lines highlight the leading edge of the cells. (F and G) Fluorescence intensity of pMLC (F) and F-actin (G) in cells treated with 500 µM H_2_O_2_, measured from the cell edge (0 µm) into the cell center (10 µm). (H) pMLC/F-actin fluorescence intensity ratio in cells treated with 500 µM H_2_O_2_, measured from the cell edge (0 µm) into the cell center (10 µm). In (F-H), the data shown represent one experiment and are averaged from at least 13 cells for each condition. The experiment was repeated three times with similar results (Figure S2G).

To test this hypothesis, we next treated the cells with blebbistatin, a nonmuscle myosin II ATPase inhibitor [40], and analyzed by qFSM the F-actin dynamics in the protrusion. Kymographs analysis of F-actin velocity in control cells (Video S4) revealed a fast F-actin retrograde flow rate in the lamellipodium (1.1 µm/min) and a slow actin retrograde flow in the lamella (0.6 µm/min) (Figure 8A-leftmost panel, B and C) as previously observed (Figure 2). Cells treated with blebbistatin for 30 min exhibited a reduced retrograde flow at 5 µm from the leading edge (0.16 µm/min) while the actin retrograde flow at the cell edge was not affected (1.1 µm/min) compared to control cells (Figure 8A-middle panel, B and C; Video S7). This result was consistent with previous studies that described the lamellipodium F-actin retrograde flow as myosin II-independent while the lamella F-actin retrograde flow is myosin II-sensitive [17], [26]. In addition, cells treated with blebbistatin and H_2_O_2_ for 30 min displayed a significant increase of the actin retrograde flow at the cell leading edge (1.6 µm/min) (Figure 8A-rightmost panel, B; Video S8) compared to unstimulated cells or cells treated with blebblistatin alone (1.1 µm/min) (Figure 8B). Of note, the F-actin flow rate at the leading edge of cells treated with blebblistatin and H_2_O_2_ was similar to cells treated with H_2_O_2_ alone (1.75 µm/min). Thus the region of fast F-actin flow at the leading edge upon H_2_O_2_ stimulation is myosin II-independent, supporting its definition as a lamellipodium. Interestingly however, the actin retrograde flow at 5 µm from the edge decreased by 6.5 fold (0.17 µm/min) in cells treated with blebbistatin and H_2_O_2_ (Figure 8A-rightmost panel, C) compared to cells treated with H_2_O_2_ alone (1.1 µm/min). Spatially resolved maps of F-actin flow rates (Figure 8A) confirmed that upon H_2_O_2_ treatment, blebbistatin abolished the formation of the wide area of fast retrograde flow further within the protrusion (Figure 8A-rigthmost panel compared to Figure 2C-bottom panel). These results indicate that, in H_2_O_2_ stimulated cells, the region of fast F-actin flow further away from the leading edge is myosin II-sensitive and thus correspond to an increase in the lamella F-actin dynamics.

To confirm these data, we measured the width of the lamellipodium in all the conditions. The results shown in Figure 8D indicated a band of 1.8 µm in cells treated with blebbistatin and H_2_O_2_ as compared to a region of 0.8 µm in control cells or cells treated with blebblistatin alone. Interestingly, the width of the lamellipodium in cells treated with blebbistatin and H_2_O_2_ was similar to the area depleted in myosin IIA after 30 min of H_2_O_2_ treatment (Figure 7A–D). Thus, H_2_O_2_ treatment induced an expansion of the lamellipodium of 2.2 fold from the cell leading edge. Taken together, these results suggest that the formation of the broad region of fast F-actin flow upon H_2_O_2_ stimulation results from an increase of the F-actin retrograde flow both in the lamellipodium and in the lamella.

Since the F-actin flow rate was enhanced in the lamella during H_2_O_2_ stimulation, we next determined whether this was due to an increase of the cell contractility in this region. Therefore, we examined by immunofluorescence the localization of serine 19-phosphorylated myosin II regulatory light chain (pMLC), an indicator of myosin II activity [41]. In control cells and in cells stimulated for 15 min with H_2_O_2_, pMLC was distributed throughout the cell. At 30–60 min, we observed a net decrease of pMLC localization at the cell leading edge (Figure 8E), confirming the expansion of the lamellipodium upon H_2_O_2_ stimulation. As mentioned above, the fluorescence intensity of F-actin was slightly enhanced during the time of stimulation compared to control cells (Figure 8G). Interestingly, we observed an increase of pMLC in the lamella of H_2_O_2_-treated cells. These observations were confirmed by quantification of the pMLC/F-actin fluorescence intensity ratio indicating a ∼1.3 fold increase at 5 µm from the leading edge in cells stimulated for 15–30 and 45 min compared to control cells (Figure 8F–H and Figure S2G). These results indicate that H_2_O_2_ treatment increases the F-actin retrograde flow in the lamella by activating the contractile machinery of the cells.

## Discussion

Productive protrusions allowing motile cells to sense and migrate toward a chemotactic gradient require a tight control of the actin cytoskeleton. Over the past years, substantial evidences have been accumulated involving ROS in the regulation of the actin network reorganization [11], [42], [43], [44]. However, the precise mechanisms of how ROS affect cell protrusion are not well elucidated yet. Detailed analysis of the spatio-temporal regulation of actin dynamics relies on the use of state-of-the art microscopy techniques, such as fluorescent speckle microscopy. One limitation of this technique is the requirement for the cell lines to form flat protrusions during the migration process, excluding neutrophils. To study the effects of ROS on cell migration and dynamics of the cell leading edge, we thus used PtK1 cells in which F-actin organization, kinetics, and kinematics have been extensively characterized. To validate the choice of PtK1 cells, we examined their response to an external source of ROS. Consistent with the studies discussed above, we showed that the presence of H_2_O_2_ modulated the migration of PtK1 cells by increasing their directionality. In addition, ROS stimulation in these cells induced the formation of a persistent protrusion.

Signals that promote cell motility are often correlated with increased cofilin activity, while those inhibiting motility enhance cofilin phosphorylation [16], [45]. Cofilin is a crucial mediator of actin cytoskeletal dynamics: it severs F-actin filaments, generating substrates for Arp2/3-mediated branching activity, and promotes F-actin depolymerization, leading to the recycling of actin monomers at the leading edge for further polymerization [16], [19], [36], [45], [46], [47], [48], [49]. Cofilin has been shown to control the directionality of motile cell in a nonmetastatic tumor cell line (MTC) and in fibroblasts [50], [51]. Furthermore, recent studies reported that ROS induced the activation of cofilin (i.e. dephosphorylation) in HeLa or VSMC [25], [52], [53] leading to an increase of cell migration. In contrast, chemotaxis was decreased in oxidatively stressed human T cells where oxidized cofilin was dephosphorylated but did not mediate F-actin depolymerization [54]. In our study, we observed a significant increase of cofilin activation during H_2_O_2_ stimulation that correlates with enhanced Arp2/3 localization at the cell edge, increased free barbed ends formation, and faster actin retrograde flow in the lamellipodium, as previously described [26]. Conversely, inhibition of ROS has been shown to block actin assembly and reduce actin retrograde flow in growth cones [55].

The precise spatio-temporal regulation of cofilin activity, as well as its local concentration within the cell leading edge are critical for cell migration [56], [57]. Studies in vitro revealed that active cofilin at low concentration leads to severed actin filaments; at higher concentration, active cofilin binds cooperatively to actin filaments without severing them; finally a very high concentration of active cofilin induces de novo nucleation that results in actin filament assembly [56]. In vivo, this can be complicated as levels of active cofilin can vary from cell-to-cell and depend on stimuli. H_2_O_2_ treatment in PtK1 cells induced an increase of active cofilin that correlates with faster F-actin turnover at the cell edge and results in a persistent protrusion. Interestingly, overexpression of active cofilin S3A in PtK1 cells was shown to decrease protrusion efficiency even though F-actin treadmilling was enhanced [26]. Altogether, these results may be explained by the relative difference in the concentration of local active cofilin in cells. Upon H_2_O_2_ stimulation, our results suggest that the cells have achieved a balance of cofilin activities leading to well structured actin networks and efficient chemotactic cells. Overexpression of active cofilin, however, induced a decoupling of lamellipodium/lamella actin networks and affected the protrusion efficiency.

Other signaling pathways downstream of ROS may also be involved in the regulation of actin dynamics. Among them, the ERK-MAPK pathway has been implicated as a signaling cascade by which cells react to H_2_O_2_ [58], [59], [60]. Importantly, recent studies have discovered that active ERK at the cell edge is critical for cell migration [38]. ERK controls the productive advancement of the cell edge by activating WAVE2 regulatory complex, required for the activation of Arp2/3 complex and subsequent actin polymerization. Our study reports an increase of active ERK at the leading edge after H_2_O_2_ stimulation and this activation is involved in the formation of a persistent protrusion. In addition, we showed that ERK activity contributes to the recruitment of Arp2/3 at the cell edge which may then works in synergy with cofilin [34], [35], [36] to increase the F-actin retrograde flow in the lamellipodium of H_2_O_2_-treated cells. This finding is particularly interesting as a recent work in zebrafish larvae has demonstrated that H_2_O_2_ induces direct oxidation-mediated activation of Lyn, a member of Src-family kinases, which in turn activates ERK at the cell edge of neutrophils [61]. This event appears to be critical to mediate leukocyte migration toward wounds in response to H_2_O_2_. Since the activity of p38 and JNK, two MAPK family members involved in the regulation of actin dynamics [62], [63], is increased upon H_2_O_2_ treatment in PtK1 cells (Figure S3F–G), it would be interesting to further investigate their contribution in protrusion dynamics.

Cell motility requires the coordination of the lamellipodium and lamella networks. Ponti et al. suggested that the lamella is necessary for the cells to migrate whereas the lamellipodium could serve as an exploratory function [17]. Interestingly, in addition to the effect of H_2_O_2_ on the lamellipodium, we also observed an increase of the actin retrograde flow in the lamella that coincides with enhanced myosin contractility. Importantly, several regulators of the contractile apparatus are ROS sensitive, which might explain the increase of MLC phosphorylation observed in the protrusion of H_2_O_2_-treated cells. The Rho/ROCK (Rho-associated protein kinase) pathway is activated by ROS in both aorta and VSMC, thus promoting MLC phosphorylation by inhibiting the regulatory subunit of MLC phosphatase [64], [65]. In addition, Pak, another major regulator of myosin contractility that can directly phosphorylate MLC [66], is activated by ROS [67]. The increased actin retrograde flow in the lamella observed upon H_2_O_2_ stimulation could thus result from Pak activation.

Our current study indicates that H_2_O_2_ increases the protrusion efficiency by modulating both the lamellipodium and lamella. Interestingly, we have recently shown that Paks regulate cell migration by modulating F-actin retrograde flow in both networks [27]. Further investigation to identify the direct targets of H_2_O_2_ controlling the molecular mechanisms that regulate the two actin networks will provide a better understanding of the role of ROS in cell migration.

## Materials and Methods

### Cell Culture

PtK1 cells (from Clare Waterman-Storer, National Institutes of Health, Bethesda, [68]) were cultured in Ham’s F12 medium (Cellgro, Manassas, VA) containing 25 mM Hepes (Gibco, Grand Island, NY), 10% FBS (Gemini Bio-Products, Sacramento, CA), 100 U/ml penicillin and 0.1 mg/ml streptomycin at 37°C in 5% CO_2_. Before H_2_O_2_ treatment, cells were starved overnight in 0.5% FBS-containing medium. For FSM experiments, cells were starved for ∼3 h after injection.

### Chemotaxis Assay

Using a microinjection system (Eppendorf), PtK1 cells were exposed or not to a constant flow of H_2_O (control) or 1.5 mM H_2_O_2_ mixed with rhodamine dextran (0.5 mg/ml). Phase-contrast time-lapse image series were acquired at 10 s intervals for 45 min using a 10X/0.25 phase objective lens (Olympus) on an inverted microscope (Olympus IX70) equipped with a Cool SNAP HQ camera. Images of rhodamine dextran were acquired every 2 min to localize the flow and determine which cells are exposed to the flow (Flow). Cells present in an area not exposed to the flow (No flow) were considered as controls. Movements of individual cells were tracked using MetaMorph software to calculate velocity and motility parameters.

### Phase-contrast Microscopy

PtK1 cells were plated on coverslips three days prior to experiments. Cells were starved overnight and maintained on the microscope stage at 37°C. Culture medium containing 0.5% FBS was added for 15 min and then cells were incubated for 45 min with control medium (+H_2_O or DMSO), with medium containing 500 µM H_2_O_2_ alone or in combination with 5 mM sodium pyruvate (Sigma-Aldrich, St. Louis, MO) or 10 µM UO126 (Cell Signaling, Beverly, MA). To analyze the effect of ROS scavenger on H_2_O_2_-induced cell protrusion, cells pretreated for 30 min with 5 mM sodium pyruvate-containing media were then maintained for 15 min in the same media and treated for 45 min with 500 µM H_2_O_2_+ROS scavenger. Phase-contrast time-lapse image series were acquired at 20 s intervals using a 20X(X1.5) phase objective lens on an inverted microscope (Eclipse TE 2000-U, Nikon). For kymograph analysis of leading edge behavior, five randomly placed lines normal to the cell edge were used. Protrusion width, persistence and protrusion-retraction velocities were calculated from these kymographs.

### Immunofluorescence Microscopy

Starved cells were treated for 0 to 60 min with 500 µM H_2_O_2_ and for 30 min with 500 µM H_2_O_2_ alone or in combination with 10 µM UO126 and fixed on coverslips with PBS containing 4% paraformaldehyde, permeabilized in PBS containing 0.5% Triton X-100 and blocked with 2% BSA in PBS. Cells were then immunolabeled for the following antibodies: myosin IIA heavy chain (Sigma-Aldrich, St. Louis, MO), pMLC (Ser^19^, a gift from Y. Sasaki, Kitasato University, Tokyo, Japan), long isoforms of tropomyosin (TM311, Sigma-Aldrich, St. Louis, MO), p34-Arc/ARPC2 (Millipore, Bedford, MA), phosphorylated-cofilin (P-cofilin, a gift from J. Bamburg, Colorado State University, Fort Collins, CO), phosphorylated-ERK (9101, Cell Signaling, Beverly, MA) by using the appropriate Alexa Fluor 488-conjugated secondary antibodies (Molecular Probes, Eugene, OR). F-actin was detected by using Alexa Fluor 568-conjugated phalloidin (Molecular Probes, Eugene, OR). Cells were mounted on slides with ProLong Gold antifade reagent (Invitrogen, Carlsbad, CA). To localize and quantify the relative number of actin filament free barbed ends, live cells were permeabilized with 0.25 mg/ml saponin in the presence of 0.5 µM X-rhodamine actin and fixed as previously described [69]. Epifluorescence images of fixed cells were acquired on an inverted microscope (Eclipse TE 2000-U, Nikon) equipped with an electronically controlled shutter, filter wheels, and a 14-bit cooled CCD camera (Cool SNAP HQ, Photometrics) controlled by MetaMorph software (Universal Imaging Corp.) with a 60X/1.4 NA Plan Apo DIC objective lens (Nikon).

### Immunofluorescence Analysis

Quantification of the fluorescence of myosin IIA, pMLC, tropomyosin, p34-Arc, P-cofilin, free barbed ends, pERK and F-actin as a function of the distance from the cell edge was obtained with custom software written in Matlab (MathWorks). Bands of constant distance to the cell edge were constructed, and individual fluorescence intensities were accumulated and averaged in each band to produce graphs representing fluorescence intensity versus distance from the leading edge (see Figure S1 for details).

### Fluorescent Speckle Microscopy

PtK1 cells were plated on coverslips three days prior to experiments and X-rhodamine-conjugated actin, prepared as described previously [68], was injected into cells at 1 mg/ml. Cells were starved for ∼3 h after injection and incubated for ∼30 min in control- or H_2_O_2_ 500 µM-containing medium in which oxyrase (Oxyrase Inc., Mansfield, OH) has been added to inhibit photobleaching. For myosin IIA inhibition, cells were incubated in culture medium containing 50 µM blebbistatin (Calbiochem, Philadelphia, PA) or blebbistatin + H_2_O_2_ 500 µM for 30 min. Actin fluorescent speckle microscopy time-lapse series were acquired at 10 s intervals for 10 min using a 60X(X1.5)/1.49 NA Plan Apo objective lens (Nikon) on a spinning disk confocal microscope system equipped with a Cool SNAP HQ camera.

### Image Analysis and Quantification

F-actin flow rates at the leading edge of the cells were measured by kymograph analysis as previously described [70]. Five randomly placed lines normal to the cell edge were used to construct kymographs for each cell. FSM time-lapse image series were analyzed using the fsmCenter software package written in Matlab (MathWorks) which allows the visualization of F-actin turnover maps [18], [71] and F-actin flow maps. To determine the width of the lamellipodium, we used the kymographs and measured the distance of the region of fast F-actin flow at the leading edge which corresponds to the lamellipodium.

### Western Blot

PtK1 cells were serum-starved overnight and then treated for 0 to 60 min with H_2_O_2_ 500 µM alone or in combination with 5 mM sodium pyruvate (Sigma-Aldrich, St. Louis, MO) or 10 µM UO126 (Cell Signaling, Beverly, MA). In the case of sodium pyruvate treatment, cells were pretreated for 30 min before addition of H_2_O_2_. The cells were lysed in RIPA buffer (50 mM Tris pH 7.4, 150 mM NaCl, 1% NP-40, 0.25% sodium deoxycholate, 1 mM EDTA) supplemented with 1 mM of aprotinin, leupeptin, pepstatin and phenylmethylsulfonyl fluoride (PMSF). 1 mM sodium orthovanadate and phosphatase inhibitor cocktail 1 (Sigma-Aldrich, St. Louis, MO) were added to this buffer for ERK, phospho-ERK, p38, phospho-p38 and phospho-JNK Western blotting. The extracts were clarified by centrifugation at 16,000×g at 4°C, and the protein concentration was estimated using the BCA protein assay (Pierce, Rockford, IL) according to the manufacturer’s instructions. Twenty micrograms of proteins were analyzed by SDS-PAGE and Western blotting using antibodies against cofilin (ACFL02, Cytoskeleton, Denver, CO), phospho-cofilin (3311, Cell Signaling, Beverly, MA), ERK (9102, Cell Signaling, Beverly, MA), phospho-ERK (9101, Cell Signaling, Beverly, MA), p38 (9212, Cell Signaling, Beverly, MA), phospho-p38 (9211, Cell Signaling, Beverly, MA), phospho-JNK (9251, Cell Signaling, Beverly, MA) and actin (clone C4, Millipore, Bedford, MA). Phospho-cofilin, phospho-ERK, phospho-p38 and phospho-JNK levels were normalized for total cofilin, ERK, p38 and actin respectively, by densitometric analysis.

### H_2_O_2_ Detection in Living Cells

The presence of ROS in the cells was monitored with PY1-AM (Peroxy-Yellow 1 Acetoxymethyl-ester), a selective fluorescent indicator for H_2_O_2_ which has been provided by C. J. Chang (University of California, Berkeley). PtK1 cells plated on glass bottom dishes were incubated with 5 µM PY1-AM in Hank’s balanced salt solution, HBSS (Invitrogen, Carlsbad, CA) for 30 min at 37°C, washed once with HBSS and treated with 500 µM H_2_O_2_ for 20 min at 37°C. Then, PY1-AM fluorescence was observed with an inverted microscope (Eclipse TE 2000-U, Nikon) using a 40X/1.4 NA Plan Apo Ph3 objective lens (Nikon).

### Statistical Analysis

Statistical analyses presented were determined using two-tailed Student’s t test.

## Supporting Information

Figure S1 **Method of immunofluorescence analysis.** Quantification of the fluorescence of P-cofilin, free barbed ends, p34-Arc, pERK, myosin IIA, tropomyosin, pMLC and F-actin as a function of the distance from the leading edge was obtained with custom software written in Matlab (MathWorks) as explained below with the example of P-cofilin (H_2_O_2_-0 min). (A) Individual fluorescence intensities of P-cofilin (left panel) and F-actin (right panel) were measured from the cell edge (0 µm) into the cell center (10 µm) for 18 cells. (B) These fluorescence intensities were then averaged and are presented with the corresponding standard deviation. (C) The ratio between the averaged P-cofilin and F-actin intensities was calculated and plotted against distance from the leading edge. This curve corresponds to the fluorescence intensity ratio P-cofilin/F-actin after 0 min of H_2_O_2_ treatment (D, left panel, black curve) for the first experiment which is shown in Figure 3D. The same process has been used for the other stimulation times (D, left panel, colored curves) and for two other independent experiments (D, center and right panels).
(TIF)

Figure S2 **Summary of immunofluorescence analysis.** The fluorescence intensity ratio P-cofilin/F-actin (A), free barbed ends/F-actin (B), p34-Arc/F-actin (C and H), pERK/F-actin (D), myosin IIA/F-actin (E), tropomyosin/F-actin (F) and pMLC/F-actin (G) were normalized to H_2_O_2_-0 min from the cell edge (0 µm) into the cell center (10 µm) for all the stimulation times in each experiment. Each curve represents averaged values from three independent experiments for H_2_O_2_ 0-15-30-45-60 min stimulation. Error bars represent s.e.m. (A) *, p<0.001 from 0 to 10 µm from the leading edge: H_2_O_2_ 15-30-45-60 min compared to H_2_O_2_-0 min. (B) *, p<0.05 from 0 to 1 µm: H_2_O_2_ 15 min and **, p<0.05 from 0 to 2 µm: H_2_O_2_ 30-45-60 min compared to H_2_O_2_-0 min. (C) *, p<0.05 from 0 to 1.6 µm: H_2_O_2_ 15-30-45-60 min compared to H_2_O_2_-0 min. (D) *, p<0.05 from 0 to 5.8 µm: H_2_O_2_ 15-30-45-60 min compared to H_2_O_2_-0 min. (E) *, p<0.05 from 0 to 4.6 µm: H_2_O_2_ 15-30-45-60 min compared to H_2_O_2_-0 min. (F) *, p<0.05 from 0 to 0.5 µm: H_2_O_2_ 15 min and **, p<0.05 from 0 to 2.6 µm: H_2_O_2_ 30-45-60 min compared to H_2_O_2_-0 min. (G) *, p<0.05 from 0 to 1.3 µm: H_2_O_2_ 30-45-60 min and **, p<0.05 from 4.6 to 6.4 µm: H_2_O_2_ 15-30-45 min compared to H_2_O_2_-0 min. (H) *, p<0.05 from 0 to 0.8 µm: H_2_O_2_ 30 min and H_2_O_2_+UO126 30 min compared to control.
(TIF)

Figure S3 **ERK activation contributes to Arp2/3 recruitment at the leading edge upon H_2_O_2_ stimulation.** (A) Cell lysates from starved PtK1 cells treated with 500 µM H_2_O_2_ and 10 µM UO126 for 0-15-30-45-60 min were immunoblotted with antibodies against pERK and ERK. (B) Immunolocalization of p34-Arc (green) and F-actin phalloidin staining (red) in starved PtK1 cells (control), and cells treated with 500 µM H_2_O_2_ or with 500 µM H_2_O_2_ and 10 µM UO126 for 30 min. The scale bar is 10 µm. (C and D) Fluorescence intensity of p34-Arc (C) and F-actin (D) in untreated cells (control), and cells treated with 500 µM H_2_O_2_ or with 500 µM H_2_O_2_ and 10 µM UO126 for 30 min, measured from the cell edge (0 µm) into the cell center (10 µm). (E) p34-Arc/F-actin fluorescence intensity ratio measured from the cell edge (0 µm) into the cell center (10 µm). In (C)–(E), the data shown represent one experiment and are averaged from at least 15 cells for each condition. The experiment was repeated three times with similar results (Figure S2H). (F) Cell lysates from starved PtK1 cells treated with 500 µM H_2_O_2_ for 0-15-30-45-60 min were immunoblotted with antibodies against phosphorylated p38 (P-p38), p38, phosphorylated JNK (P-JNK) and actin. In (G), the graphs represent the averaged normalized P-p38 and P-JNK values. Data are from three independent experiments. Error bars represent s.e.m. *, p<0.05 and **, p<0.01 compared to H_2_O_2_-0 min (G).
(TIF)

Video S1 **Phase-contrast imaging of migrating PtK1 cells under control conditions.** Epithelial PtK1 cells were incubated for 15 min in media only and then treated for 45 min with control media containing H_2_O. The black frame indicates the beginning of the treatment with the control media. Images were acquired at 20 s intervals on an inverted microscope.
(MOV)

Video S2 **Phase-contrast imaging of migrating PtK1 cells stimulated with H_2_O_2_.** Epithelial PtK1 cells were incubated for 15 min in media only and then treated for 45 min with media containing 500 µM H_2_O_2_. The black frame indicates the beginning of the treatment with 500 µM H_2_O_2_. Images were acquired at 20 s intervals on an inverted microscope.
(MOV)

Video S3 **Phase-contrast imaging of migrating PtK1 cells treated with H_2_O_2_+ROS scavenger.** Epithelial PtK1 cells pretreated with media containing ROS scavenger (5 mM) were incubated for 15 min in the same media and then treated for 45 min with media containing 500 µM H_2_O_2_+ROS scavenger. The black frame indicates the beginning of the treatment with 500 µM H_2_O_2_+ROS scavenger. Images were acquired at 20 s intervals on an inverted microscope.
(MOV)

Video S4 **F-actin FSM of a PtK1 cell under control conditions.** Epithelial PtK1 cells were injected with X-rhodamine actin and incubated in control media. Images were acquired on a spinning-disk confocal microscope every 10 s for 10 min.
(MOV)

Video S5 **F-actin FSM of a PtK1 cell stimulated with H_2_O_2_.** Epithelial PtK1 cells were injected with X-rhodamine actin and treated with 500 µM H_2_O_2_. Images were acquired on a spinning-disk confocal microscope every 10 s for 10 min.
(MOV)

Video S6 **Phase-contrast imaging of migrating PtK1 cells treated with H_2_O_2_+UO126.** Epithelial PtK1 cells were incubated for 15 min in media only and then treated for 45 min with media containing 500 µM H_2_O_2_+10 µM UO126, a MEK inhibitor. The black frame indicates the beginning of the treatment with 500 µM H_2_O_2_ and 10 µM UO126. Images were acquired at 20 s intervals on an inverted microscope.
(MOV)

Video S7 **F-actin FSM of a PtK1 cell treated with blebbistatin.** Epithelial PtK1 cells were injected with X-rhodamine actin and treated with 50 µM blebbistatin for 30 min. Images were acquired on a spinning-disk confocal microscope every 10 s for 5 min.
(MOV)

Video S8 **F-actin FSM of a PtK1 cell treated with blebbistatin + H_2_O_2_.** Epithelial PtK1 cells were injected with X-rhodamine actin and treated with 50 µM blebbistatin+500 µM H_2_O_2_ for 30 min. Images were acquired on a spinning-disk confocal microscope every 10 s for 5 min.
(MOV)
