# Supplementary figures and images for: Reactive Oxygen Species Regulate Protrusion Efficiency by Controlling Actin Dynamics
Source: PLoS One. 2012 Aug 2;7(8):e41342. doi: 10.1371/journal.pone.0041342 (PMC3410878; doi:10.1371/journal.pone.0041342)

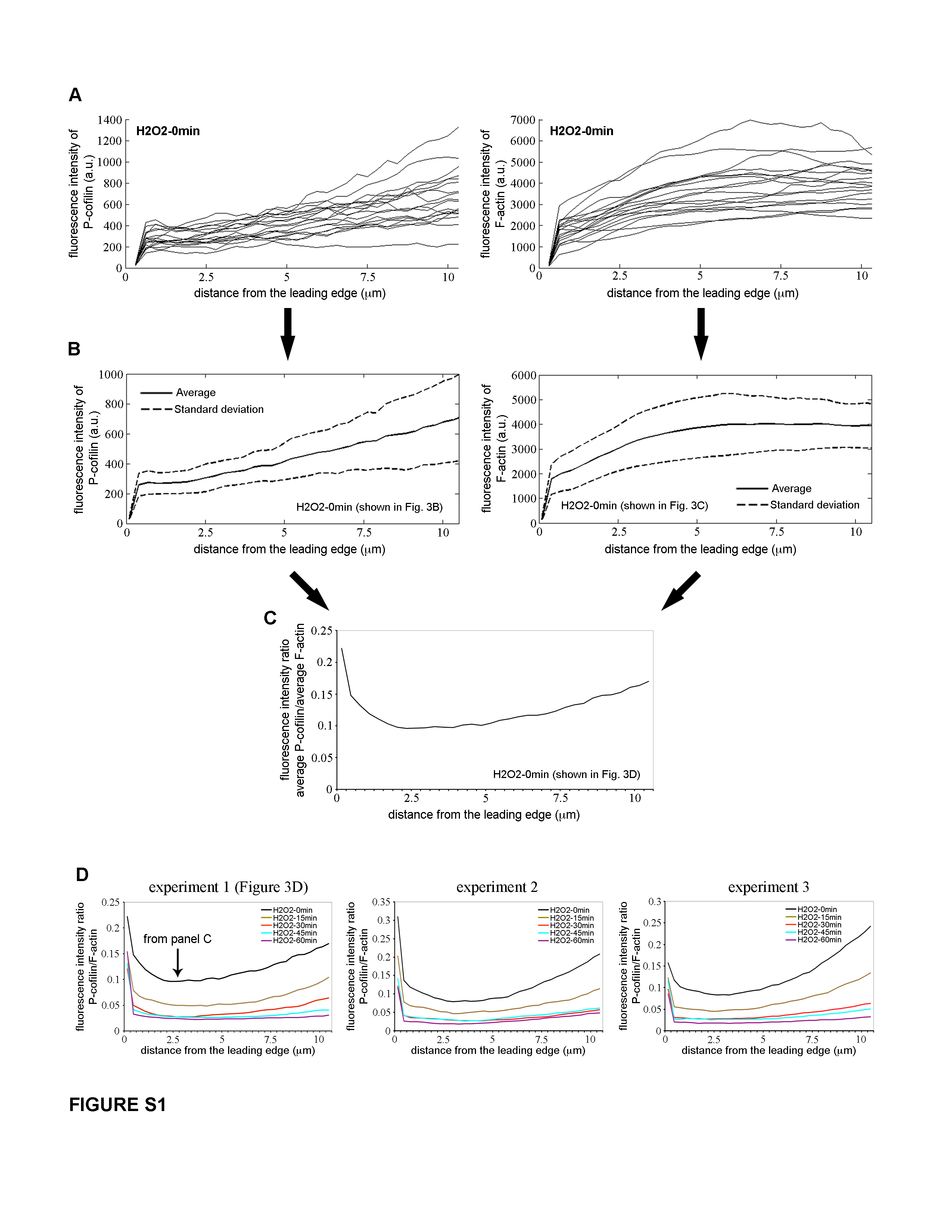

Supplement: Figure S1 — Method of immunofluorescence analysis. Quantification of the fluorescence of P-cofilin, free barbed ends, p34-Arc, pERK, myosin IIA, tropomyosin, pMLC and F-actin as a function of the distance from the leading edge was obtained with custom software written in Matlab (MathWorks) as explained below with the example of P-cofilin (H2O2-0 min). (A) Individual fluorescence intensities of P-cofilin (left panel) and F-actin (right panel) were measured from the cell edge (0 µm) into the cell center (10 µm) for 18 cells. (B) These fluorescence intensities were then averaged and are presented with the corresponding standard deviation. (C) The ratio between the averaged P-cofilin and F-actin intensities was calculated and plotted against distance from the leading edge. This curve corresponds to the fluorescence intensity ratio P-cofilin/F-actin after 0 min of H2O2 treatment (D, left panel, black curve) for the first experiment which is shown in Figure 3D. The same process has been used for the other stimulation times (D, left panel, colored curves) and for two other independent experiments (D, center and right panels). (TIF) [file pone.0041342.s001.tif]

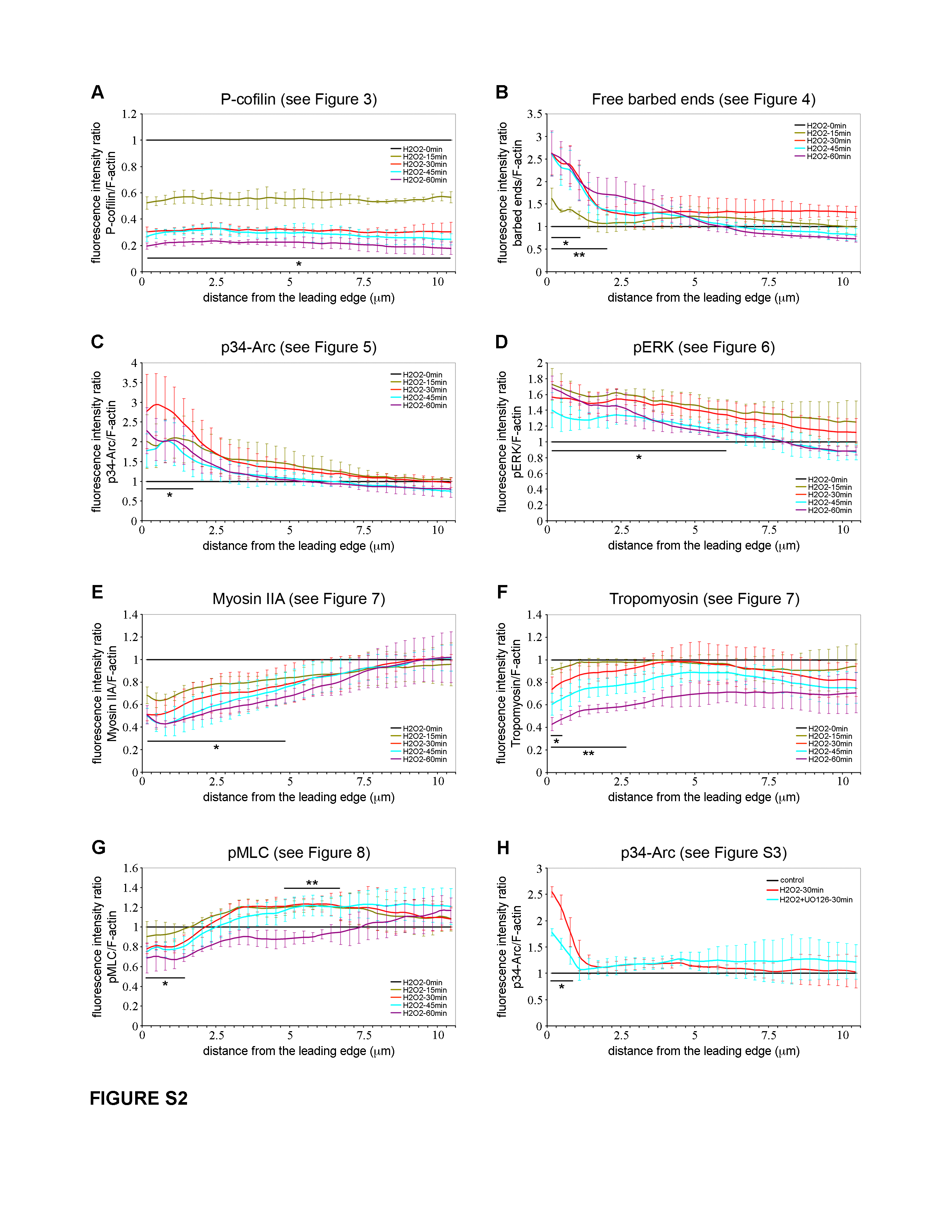

Supplement: Figure S2 — Summary of immunofluorescence analysis. The fluorescence intensity ratio P-cofilin/F-actin (A), free barbed ends/F-actin (B), p34-Arc/F-actin (C and H), pERK/F-actin (D), myosin IIA/F-actin (E), tropomyosin/F-actin (F) and pMLC/F-actin (G) were normalized to H2O2-0 min from the cell edge (0 µm) into the cell center (10 µm) for all the stimulation times in each experiment. Each curve represents averaged values from three independent experiments for H2O2 0-15-30-45-60 min stimulation. Error bars represent s.e.m. (A) *, p<0.001 from 0 to 10 µm from the leading edge: H2O2 15-30-45-60 min compared to H2O2-0 min. (B) *, p<0.05 from 0 to 1 µm: H2O2 15 min and **, p<0.05 from 0 to 2 µm: H2O2 30-45-60 min compared to H2O2-0 min. (C) *, p<0.05 from 0 to 1.6 µm: H2O2 15-30-45-60 min compared to H2O2-0 min. (D) *, p<0.05 from 0 to 5.8 µm: H2O2 15-30-45-60 min compared to H2O2-0 min. (E) *, p<0.05 from 0 to 4.6 µm: H2O2 15-30-45-60 min compared to H2O2-0 min. (F) *, p<0.05 from 0 to 0.5 µm: H2O2 15 min and **, p<0.05 from 0 to 2.6 µm: H2O2 30-45-60 min compared to H2O2-0 min. (G) *, p<0.05 from 0 to 1.3 µm: H2O2 30-45-60 min and **, p<0.05 from 4.6 to 6.4 µm: H2O2 15-30-45 min compared to H2O2-0 min. (H) *, p<0.05 from 0 to 0.8 µm: H2O2 30 min and H2O2+UO126 30 min compared to control. (TIF) [file pone.0041342.s002.tif]

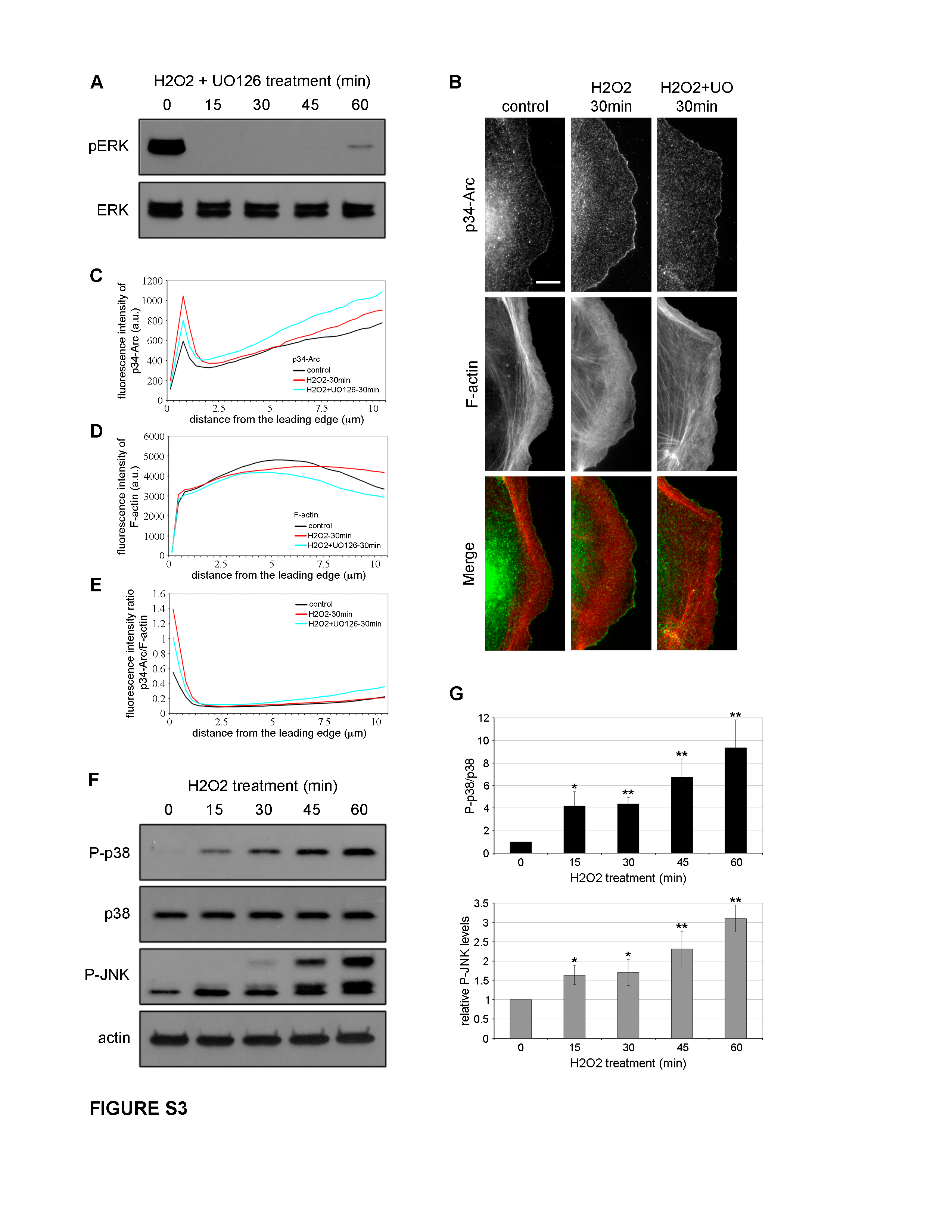

Supplement: Figure S3 — ERK activation contributes to Arp2/3 recruitment at the leading edge upon H2O2 stimulation. (A) Cell lysates from starved PtK1 cells treated with 500 µM H2O2 and 10 µM UO126 for 0-15-30-45-60 min were immunoblotted with antibodies against pERK and ERK. (B) Immunolocalization of p34-Arc (green) and F-actin phalloidin staining (red) in starved PtK1 cells (control), and cells treated with 500 µM H2O2 or with 500 µM H2O2 and 10 µM UO126 for 30 min. The scale bar is 10 µm. (C and D) Fluorescence intensity of p34-Arc (C) and F-actin (D) in untreated cells (control), and cells treated with 500 µM H2O2 or with 500 µM H2O2 and 10 µM UO126 for 30 min, measured from the cell edge (0 µm) into the cell center (10 µm). (E) p34-Arc/F-actin fluorescence intensity ratio measured from the cell edge (0 µm) into the cell center (10 µm). In (C)–(E), the data shown represent one experiment and are averaged from at least 15 cells for each condition. The experiment was repeated three times with similar results (Figure S2H). (F) Cell lysates from starved PtK1 cells treated with 500 µM H2O2 for 0-15-30-45-60 min were immunoblotted with antibodies against phosphorylated p38 (P-p38), p38, phosphorylated JNK (P-JNK) and actin. In (G), the graphs represent the averaged normalized P-p38 and P-JNK values. Data are from three independent experiments. Error bars represent s.e.m. *, p<0.05 and **, p<0.01 compared to H2O2-0 min (G). (TIF) [file pone.0041342.s003.tif]
